# Revolutionizing pediatric neurology care: telemedicine advancements and regulatory impact in the Northeast of Brazil

**DOI:** 10.1055/s-0045-1806733

**Published:** 2025-04-22

**Authors:** Daniela Laranja Gomes Rodrigues, Melina Alves da Frota, Maísa Vieira da Silva Malta, Marcos Aurélio Maeyama, Frederica Padilha, Adriana Miyauchi, Vanessa dos Santos Gonçalves Senra, André Felipe Martins de Araújo Silva, Deyse Mirelle Souza Santos, Eno Dias de Castro Filho, Nídia Cristina de Sousa, Ana Paula Marques de Pinho, Fernanda Saks Hahne

**Affiliations:** 1 Hospital Alemão Oswaldo Cruz, Responsabilidade Social, São Paulo SP, Brazil.; 2 Núcleo de Telessaúde, Florianópolis SC, Brazil.; 3 Secretaria de Saúde, Aracaju SE, Brazil.

**Keywords:** Telemedicine, Public Health Systems, Neurology, Child

## Abstract

**Background** Telemedicine, a patient-centered approach that leverages technology, has emerged as a cost-effective solution to provide comprehensive medical services for acute and chronic conditions.

**Objective** We aim to document the experiences of pediatric neurologists using teleconsultations within the TeleNortheast program, a specialized medical assistance initiative in the Northeast of Brazil.

**Methods** A retrospective analysis of teleconsultations was conducted from January to October 2023. Data from 546 teleconsultations, involving 506 pediatric patients, were extracted from medical records. Consultations were conducted between pediatric neurologists in São Paulo and primary care professionals in Sergipe. The analysis included patients' demographics, diagnoses, and outcomes.

**Results** Of the 506 teleconsultation patients seen, 89% continued treatment within their primary care settings without needing in-person referrals. Discharge was observed in 9.5% of cases, and only 1.4% required specialized in-person care. The most frequent diagnosis was autism spectrum disorder (19.1%), followed by epilepsy (7.1%), attention-deficit/hyperactivity disorder (6.7%), and intellectual disability (5.9%). A cost-saving analysis indicated that teleconsultations avoided significant transportation costs, which could reach up to R$ 21 thousand for travel and feeding expenses.

**Conclusion** The results highlight the effectiveness of teleconsultations in improving access to pediatric neurological care in underserved regions, reducing the need for in-person counseling, with the potential to provide significant cost savings for the public health system. The TeleNortheast program demonstrates the potential of this approach to bridge healthcare gaps, particularly in regions with limited access to specialized care.

## INTRODUCTION

According to the World Health Organization (WHO, 2010), 1 telemedicine is “the use of telecommunications to diagnose and treat diseases and ill-health.” This model has shown numerous benefits in the care of acute and chronic diseases for patients, families, caregivers, and health systems, as it leverages informatics strategies to deliver comprehensive and specialized medical services at reduced costs. 2 3 4

Numerous studies have validated its effectiveness, particularly in managing diseases like epilepsy, headaches, as well as movement and developmental disorders. The growing implementation of telemedicine in pediatric neurology during the COVID-19 pandemic has underscored its relevance as an essential tool in delivering continuous care. This practice has been notably beneficial in conditions like epilepsy, 5 in which telemedicine allows for remote management, reduces travel costs, and limits disruptions to family life. 6 In pediatric epilepsy, for example, this modality has shown to be a feasible and effective alternative to in-person visits, maintaining or improving seizure control and ensuring high satisfaction among caregivers.

Beyond epilepsy, telemedicine has also proven valuable in the early diagnosis and management of other neurological conditions like autism spectrum disorder (ASD) and migraines. Studies have demonstrated that this approach is not only viable for diagnosing ASD but can also be an essential part of early intervention. 7 8 This broad-based evidence emphasizes the potential of telemedicine to improve neurological care in pediatric populations globally. A more detailed literature review reveals a growing body of work supporting its effectiveness across various settings, with findings consistently pointing to its cost-efficiency, accessibility, and positive patient and caregiver satisfaction outcomes.

Virtual visits, or teleconsultations, are approaches that can be performed between patients or physicians without direct contact. Information technology facilitates these visits and safeguards user data protection measures. In Brazil, telemedicine, including teleconsultation, is validated by the resolution No 2.314/2022 of the Federal Council of Medicine, 9 which marks substantial progress in enhancing healthcare accessibility throughout the country. 10

In 2023, Brazil had a rate of 2.6 physicians per 1,000 inhabitants, 11 slightly below the Organization for Economic Co-operation and Development (OECD) average of 3.36. 12 Although this rate is growing, the distribution of doctors within the country remains uneven among states, especially among specialists, as with pediatric neurologists. 11 Furthermore, professionals tend to concentrate primarily in significant capitals, making it difficult for the countryside population to access specialized healthcare.

These adversities lead to prolonged waiting queues for appointments and impose physical and emotional burdens on patients, families, and caregivers. 13 Therefore, telehealth strategies emerge as a viable solution to address these challenges, particularly in underserved regions where healthcare professionals are limited, and transportation costs are high. Healthcare systems can overcome resource constraints and geographical barriers by leveraging these technologies, ultimately enhancing their efficiency and effectiveness. 3 14

In response to the healthcare needs of a population with limited access to it, the TeleNortheast program was designed to bridge the healthcare gap in northeastern states of Brazil, where it is more pronounced. The provision of specialties included in the program was based on the needs expressed by local managers, consideration of the queue, the waiting time, telehealth's capability, and the sensitivity of Primary Care Units to the most prevalent conditions of each specialty. This initiative is part of the Specialized Medical Assistance through Telemedicine program in the Northeast region, coordinated by the Institutional Development Support Program for the Unified Health System (IDSP-UHS) under the Brazilian Ministry of Health. 15

The program was held in Sergipe, with a human development index (HDI) of 0.70 (17 ^th^ in Brazil) and a total population of 2.2 million inhabitants in 2022, 16 divided into 75 municipalities. Information from the latest report on the medical demographic 11 demonstrates that Sergipe has a ratio of 2.15 professionals per thousand inhabitants and the worst distribution of doctors throughout the state. In the metropolitan regions (including the capital), this ratio is 13 times higher than in the other municipalities. Of the total, 63.1% are specialists. In comparison, 36.9% are general practitioners, establishing a ratio of 1.71 specialists for every general practitioner, with 91.8% of these physicians concentrated in the capital, highlighting the need for such a program in this state.

The objective of this study is to demonstrate the results of the teleconsultations conducted between the pediatric neurology team of the Hospital Alemão Oswaldo Cruz (HAOC), located in São Paulo, and the primary care physicians from the state of Sergipe, between January and October of 2023, within the TeleNortheast program. We further aim to assess this approach's importance in delivering pediatric neurological care and explore patient and provider perspectives on its use. In the **Supplementary Material** (available at https://www.arquivosdeneuropsiquiatria.org/wp-content/uploads/2024/12/ANP-2024.0080-Supplementary-Material.docx ), we estimate the distance and costs of travel to the capital for consultations. This data contributes to the debate on the potential benefits of telemedicine in Brazil.

## METHODS

This cross-sectional study utilizes retrospective data from teleconsultations conducted as part of the TeleNortheast program by the pediatric neurology team between January and October 2023. All patients scheduled for neuropediatric consultations by Primary Care Units (UBS) were included in this study. The neuropediatric team at Hospital Alemão Oswaldo Cruz (HAOC) offered specific dates and times available, which were then filled at the discretion of primary care physicians in Sergipe, Brazil.

The inclusion criteria encompassed children up to 17-years-old referred by their primary care physicians for neurological evaluation. The healthcare units included in the study were selected based on collaboration with local health authorities, prioritizing those with a significant number of neuropediatric on their waiting lists. Only units with a reliable internet connection for real-time teleconsultations were eligible for participation. Units that could not meet the technical requirements, such as stable internet connectivity or the presence of a primary care physician, were excluded.

Virtual visits were performed between primary care professionals and pediatric neurologists from HAOC. Each teleconsultation lasted a maximum of 30 minutes, and all four neuropediatric underwent prior training following institutional protocols on conducting teleconsultations effectively, following established institutional guidelines to ensure a structured and efficient consultation process. The primary care physicians were also trained to facilitate these sessions, particularly in patient preparation and follow-up coordination.

The data analysis involved retrospectively compiling relevant patient information, which was then organized and processed in Microsoft Excel (Microsoft Corp., Redmond, WA, USA) and the R (R Foundation for Statistical Computing, Vienna, Austria) software for subsequent descriptive statistical analysis, with analysis of frequencies and relative frequencies, means, and bivariate analyses. This process provided a comprehensive overview of the dataset, including patient demographics, diagnoses, and consultation outcomes, offering valuable insights into the distribution and nature of pediatric neurology cases addressed through telemedicine by the pediatric neurology team as part of the TeleNortheast program, from January to October 2023.

The data analysis process involved compiling relevant information, which was then organized and inputted into Excel for subsequent statistical analysis to provide a comprehensive overview of the dataset's distribution was obtained from the Hospital Alemão Oswaldo Cruz database and encompassed the following information: children's age at the time of consultation, gender, reason for referral to the pediatric neurologist, diagnosis, and consultation outcomes. Following each virtual visit, patients were categorized into three different groups regarding potential outcomes:

- Recommendation of a scheduled Telehealth return for further discussion with the specialist (return to primary care with teleconsultation);
- Recommendation of referral for in-person specialized care; or
- Recommendation of follow-up in primary care only.

A total of 506 individual patients were included in the study, resulting in 548 teleconsultations (506 initial and 42 follow-ups).

Per ordinance No. 2.488/200717, in cases that do not provide transportation, the patient and their companion receive an allowance of $8.40 each for food, excluding overnight stays, and $4.75 for every 50 kilometers of land travel relative.

The Institutional Review Board (IRB) of the hospital Alemão Oswaldo Cruz approved this research, with registration number 65491722.0.0000.0070.

## RESULTS

The TeleNortheast program covered 34 municipalities across the state, for which Table 1 shows some sociodemographic indicators, as well as the capital Aracaju (nonparticipant). A total of 315 Primary Care Units participated in the program, and, except for one case, all the teleconsultations were accompanied by primary care physicians, with 131 doctors attending a minimum of one consultation and a maximum of 22.

**Table 1:** Sociodemographic indicators of Sergipe municipalities covered by the TeleNortheast program

	Median	Min	Max	Aracaju
Primary care coverage (%)	95	0	100	74%
Doctors/1,000hab*	0.78	0.37	2.21	4.6
Life expectancy (in years)	70.4	66.9	73.0	74.3
Population with basic sanitation (%)	26%	1%	64%	85.3%
Population	23,139	3,309	106,015	672,614
Bolsa família coverage**	65%	44%	98%	22%
Distance to Aracaju (km)	83	21	151	

Notes: *Number of doctors per 1,000 inhabitants. ** A social welfare program in Brazil aimed at providing financial aid to low-income families. Source: Instituto de Estudos para Políticas de Saúde data (IEPSDATA).

Table 2 provides a comprehensive descriptive analysis of the participant profile in this study. Most patients were identified as male, constituting 73% of the overall cohort sample, while females composed 27%. The age distribution depicts a diverse representation. The highest frequency was observed in the 0 to 4-year-old group, encompassing 35% of participants, closely followed by the 5 to 8-year-old group (32%). Subsequent age categories include 9 to 12 (26%) and 13 to 17 (7%) years. The average age of boys was 7.3 years, while that of girls was 6.4 years. The small sample and the multiplicity of diagnoses are significant limitations to correlation analyses between patient characteristics and diagnoses. Taking this restriction into account, when we examined the 97 patients diagnosed with ASD, we observed a higher incidence in boys (22%) than in girls (11%), confirming data from the literature in the area. 17 18

**Table 2:** Patients' profiles

		Frequency	%
**Sex**	Male	368	72.7
	Female	138	27.3
**Age (in years)**	0–4	145	28.7
	5– 8	130	25.7
	9–12	103	20.4
	13–17	28	5.5
	Without data	100	19.
**Outcome**	Discharge	48	9.5
	Referral to in-person consultation	7	1.4
	Return to primary care with teleconsultation	451	89.1

Considering the outcome of the consultations for the 506 patients, 89% (n = 451) of cases, patients continued their treatment within the primary care system with further teleconsultations, indicating no need for in-person referrals. Discharge from specialist care through teleinterconsultation was recommended in 109.5% (4,852) of cases, suggesting resolution or independent management by the primary care physician improvement. Only 1.4% (7) of the cases required referral for specialized in-person consultation, demonstrating the efficacy of teleconsultations in avoiding unnecessary travel and further intervention.

**Figure 1:**
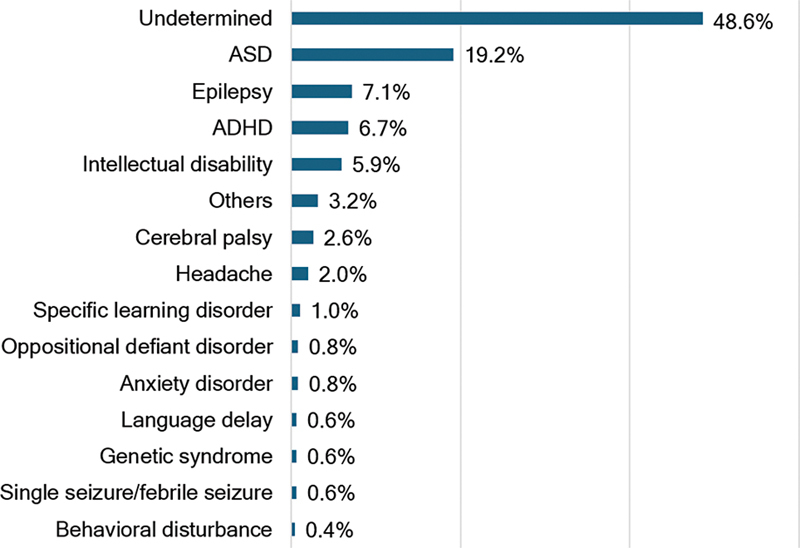
Diagnostic of the studied population.

Regarding diagnoses, 48.6% of patients had undetermined diagnoses at the time of the initial teleconsultation ( Figure 1 ). This demonstrates the complexity of the referred cases and highlights the need for follow-up consultations for further evaluation and pediatric neurological management. Among those with confirmed diagnoses, the most prevalent condition was ASD, affecting 19.2% of patients, followed by epilepsy (7.1%), Attention-Deficit/Hyperactivity Disorder (ADHD) with 6.7%, 17 18 and intellectual disability at 5.9%. Other conditions with lower prevalence included cerebral palsy (2.6%) and headache (2%). Diagnoses with lower prevalence were grouped under the “other cases” diagnostic group and included nonepileptic events, cranial nerve palsy, traumatic brain injury, neonatal anoxia, sleep disorders, and brain cysts.

In addition to the teleconsultations analyzed in this study, there were 13 appointments in which patients did not show up, resulting in an abstention rate of 2.3%, significantly lower than the rate recorded in primary care units, 19 20 which is around 25%.

## DISCUSSION

In the current study, we presented the results of teleconsultations conducted between the neuropediatric team of Hospital Alemão Oswaldo Cruz (HAOC) and primary care physicians in the Northeast region of Brazil under the TeleNortheast program.

In 89% of cases, the clinical outcome was a return to telemedicine in the primary care environment without the need to travel to an in-person consultation in another location. That demonstrates that the teleconsultation interaction tool is sensitive to neuropediatrics. Only 1.4% of cases showed the necessity of a neuropediatric neurology specialist's in-person intervention, significantly reducing waiting queues. In the remaining 9.5% of cases, there was a suggestion to discharge patients from the specialist's care and continue monitoring within primary health.

Therefore, teleconsultation offers two direct outcomes that enhance patient access to healthcare. First, it provides immediate access to care through interactions between primary care physicians and specialists, enabling prompt initiation of necessary interventions and ongoing management when required. Second, these interactions may empower primary care physicians with enhanced expertise in case management, improving diagnosis, treatment, and referral decisions without additional teleconsultations for similar cases. Consequently, access to specialists in person, typically regulated by waiting lists, is reserved for more complex and challenging cases that often need direct resolution. This process of professional development ensures the sustainability of project gains.

The diagnostic landscape found in this sample demonstrated a variety of conditions, with ASD standing out as the predominant category considering the determined diagnosis. That follows the recent increase in ASD prevalence.

The latest report from the Centers for Disease Control and Prevention monitoring studies showed a prevalence of 1 case for every 36 children in 2020. In 2000, the prevalence was 1:150 children, considering the age range of 8 years. 18 In Brazil, although there are no robust surveys determining the actual prevalence of neurodevelopmental disorders, 21 estimations, and small studies indicate the high burden of these diseases on the healthcare system, 21 22 23 underscoring the importance of further research and comprehensive data collection efforts to accurately assess the prevalence and impact of neurodevelopmental disorders in this population.

The high prevalence of neurodevelopmental disorders also contributed to the considerable number of patients with undetermined diagnoses by the time of the teleconsultation. In our experience, although many patients were referred because there were signs of ASD, in almost half of the cases, it was impossible to safely diagnose them in a single 30 minute appointment, requiring a return consultation for further analysis ( Figure 1 ). The time needed for ASD diagnosis can vary considerably depending on symptom severity, specialist availability, and access to healthcare. 24 A study on diagnostic practices in Canada estimated an average timeframe of 7 months from referral to formal diagnosis, 25 with instances where the process extends for over a year. 26

A significant proportion of patients in this study, 48.5%, remained with undetermined diagnoses after the initial teleconsultation. This finding highlights the complexity of pediatric neurological cases and the inherent challenges in reaching a definitive diagnosis in a single consultation. In conditions like ASD and epilepsy, multiple consultations are often required before a precise diagnosis can be made. For example, the diagnostic process for ASD is particularly complex, with studies showing that the time from referral to formal diagnosis can take several months and often requires follow-up consultations to observe the child's development and behavior over time. 18 Similarly, in epilepsy, although telemedicine can be effective in managing care remotely, it frequently necessitates further evaluations to confirm the diagnosis. 3 The high percentage of undetermined diagnoses underscores the necessity of follow-up consultations in telemedicine to ensure accurate diagnoses and appropriate management, particularly for complex neurodevelopmental and neurological disorders in pediatric populations.

Considering that specialists primarily operate in the state's capital, Aracaju, we face another challenge: addressing the transportation and associated user costs. In the Brazilian Unified Health System, authorities must arrange their transfer to another town for assistance when it lacks the necessary health resources for a patient. For instance, if pediatric neurologists are only available in Aracaju, patients from other cities must travel to the capital for the visit. 27 In such scenarios, their city of residence becomes responsible for covering the costs of transportation, food, and accommodation.

Considering the rates applied in the SUS, a consultation in Aracaju could have a transport cost of up to R$76.20 for patients from cities such as Garuru and Poço Verde, located 150 km from the capital ( **Supplementary Material** ). For comparison purposes, data from the National Supplementary Health Agency indicate that the average cost of a specialist consultation in the Brazilian private healthcare market is $73. In contrast, the public healthcare system pays only R$10 to states and cities to provide this service. This amount needs to be supplemented with the budget of these federative entities. Furthermore, other immaterial benefits of telehealth must be considered, such as the rapid access to specialized assessment in the primary care service itself, avoiding the risks of long car journeys, and a lower absenteeism rate.

This study contributes valuable insights into the effectiveness of teleconsultations in addressing healthcare gaps, especially in underserved regions. It is essential to acknowledge the invaluable support pediatric neurologists provide in facilitating and enhancing the quality of healthcare provided by general practitioners, who coordinate patient care. The TeleNortheast program, as part of the Specialized Medical Assistance through Telemedicine Program, 15 exemplifies a successful initiative in bridging healthcare disparities. The findings underscore the potential of telemedicine in improving accessibility, particularly for pediatric neurology services, and emphasize the need for continued efforts to develop telehealth strategies in addressing diverse needs in healthcare.

Our research has certain limitations that should be acknowledged. One fundamental limitation is that the study focuses exclusively on the Northeast region of Brazil, specifically in Sergipe, which may limit the generalizability of the findings to other areas with different demographic, socioeconomic, or health characteristics. Additionally, the study spanned a relatively brief period, from January to October 2023, potentially constraining the capture of long-term trends or seasonal variations in the use of this approach in pediatric neurology. The absence of a control group also limits the study's ability to compare telemedicine with conventional in-person approaches in pediatric neurology.

Another significant limitation is the need for more data on internet infrastructure, which plays a critical role in the success of telemedicine. In regions with poor or unstable connections, the quality of care may be compromised, leading to delayed diagnoses or miscommunication between healthcare professionals. Moreover, the present study did not account for the socioeconomic challenges patients face, such as access to technology, digital literacy, or the availability of technological resources, all of which could influence the success of teleconsultations, particularly in underserved areas.

Additionally, there needs to be more age data for approximately one hundred individuals in our dataset, a limitation inherent in the study's retrospective nature, where data was collected from preexisting medical records. These studies often need more datasets due to variable record practices, leading to occasional omissions. Although this absence limits age-based analysis, the available data remains valuable for understanding overall trends in telemedicine use. Future research should focus on evaluating these factors and addressing such data gaps, as they are essential for comprehensively understanding the impact of telemedicine on healthcare delivery.

In conclusion, the implementation of the Brazilian resolution 2.314/2022 marks a significant advancement in telemedicine by establishing a robust regulatory framework that supports various telehealth strategies, including teleconsultations. This regulatory initiative has notably enhanced healthcare access in regions with limited resources, particularly in underserved areas such as Sergipe. The study emphasizes the success of teleconsultations between the pediatric neurology team at Hospital Alemão Oswaldo Cruz in São Paulo and primary care physicians in Sergipe, demonstrating the efficient resolution of such cases.

Furthermore, beyond the economic benefits, the study highlights immaterial advantages such as reducing long-distance travel, providing timely access to specialists, and improving care coordination. These aspects reduce costs and waiting times and foster strong collaboration between primary and specialized care, resulting in more efficient treatments and fewer missed appointments.

While this study focused on Sergipe, the results have broader implications. The telemedicine model for pediatric neurology can be extended to other regions in Brazil facing similar healthcare access challenges. Moreover, this approach is adaptable to other medical specialties like cardiology and psychiatry, where shortages of specialists and the need for long travel distances to receive care are significant barriers. Integrating telemedicine into various specialties allows the public health system to bridge healthcare gaps further, ensuring more equitable and timely care for underserved populations nationwide.

These findings confirm telemedicine's potential to reshape healthcare delivery in Brazil. It offers economic and qualitative improvements that enhance care efficiency and patient outcomes across multiple specialties.
